# Toxicological evaluation of *Terminalia paniculata* bark extract and its protective effect against CCl_4_-induced liver injury in rodents

**DOI:** 10.1186/1472-6882-13-127

**Published:** 2013-06-06

**Authors:** Sahil Talwar, Hitesh V Jagani, Pawan G Nayak, Nitesh Kumar, Anoop Kishore, Punit Bansal, Rekha R Shenoy, Krishnadas Nandakumar

**Affiliations:** 1 Department of Pharmacology, Manipal College of Pharmaceutical Sciences,Manipal University, Manipal, Karnataka 576 104, India; 2 Department of Pharmaceutical Biotechnology, Manipal College of Pharmaceutical Sciences, Manipal University, Manipal, Karnataka 576 104, India

**Keywords:** Terminalia paniculata, Antioxidant, Hepatoprotective, CCl_4_-induced liver toxicity, Mitochondrial staining, Apoptotic markers

## Abstract

**Background:**

Based on the reported antioxidant and anti-inflammatory potential of *Terminalia paniculata*, the bark aqueous extract (TPW) was investigated against liver damage.

**Methods:**

Intrinsic cytotoxicity was tested on normal human liver (Chang) cell lines, followed by acute and sub-chronic toxicity studies in mice. TPW was then evaluated against CCl_4_-induced liver toxicity in rats. Liver enzymes (AST, ALT, and ALP) and antioxidant markers were assessed. The effect of TPW on isolated hepatic cells, post-CCl_4_ administration, was assessed by isolated mitochondrial membrane staining. The actions of TPW on apoptotic pathway in CCl_4_-treated Chang cells were also elucidated.

**Results:**

TPW was found to be safe at all doses tested in both *in vitro* and *in vivo* toxicity studies. TPW (400 mg/kg, p.o.) significantly (*p <0.05) improved liver enzyme activity as compared to CCl_4_. Also, it improved antioxidant status (GSH, GST, MDA and total thiol) and preserved hepatic cell architecture. TPW pre-treatment significantly attenuated the levels of phospho-p53, p53, cleaved caspase-3, phospho-Bad, Bad and cleaved PARP in CCl_4_-treated Chang cells, improving the viability considerably.

**Conclusion:**

The findings support a protective role for *Terminalia paniculata* in pathologies involving oxidative stress.

## Background

Hepatic diseases represent a serious health problem for which modern medicine offers few effective treatments apart from traditional herbal formulations [1]. Numerous medicinal plant formulations are used to treat liver disorders in Indian ethnomedical practice and traditional therapy. Most of the treatments act as radical scavengers, whereas others are enzyme inhibitors or mitogens [2].

*Terminalia paniculata* Roth. (Combretaceae) is a tropical tree with a broad natural distribution in Western Ghats, India. Extracts prepared from the flowers and bark of *Terminalia paniculata* have been used as remedies for cholera, inflamed parotid glands and menstrual disorders in the traditional system of medicine [3]. Previous studies from our laboratory have demonstrated the presence of polyphenolics (mainly flavonoids and tannins) and provided evidence for the anti-inflammatory potential (based on its folklore use) of this plant in chronic and acute models of inflammation [4] along with potent antioxidant activity (data unpublished).

Polyphenolic compounds, which are antioxidants of natural origin, have generated considerable interest as potential therapeutic agents for a wide variety of chronic diseases. Many experiments have shown that flavonoids and tannins possess hepatoprotective effects in various experimental models [5,6].

In the present study, we performed *in vitro* cell based cytotoxicity assays in Chang liver cell lines and an *in vivo* toxicological evaluation of TPW to assess its safety profile. The carbon tetrachloride (CCl_4_)-induced hepatotoxicity model was employed to assess the protective effects of TPW *in vivo*. CCl_4_, a proven experimental agent for inducing acute liver injury [7], is biotransformed by hepatic microsomal cytochrome P450 to trichloromethyl-free radical (CCl_3_* or CCl_3_OO*) [8]. These metabolites react with antioxidant enzymes such as catalase and superoxide dismutase (SOD) and lead to lipid peroxidation and liver damage. Hepatic enzyme levels (AST, ALT, and ALP) along with endogenous antioxidant profiling (GSH, GST, MDA and total thiol) were investigated. Mitochondrial membrane staining and histopathological analysis of the liver tissues was also carried out.

## Methods

### Chemicals

MTT (3-(4,5-Dimethylthiazol-2-yl)-2,5-diphenyltetrazolium bromide), sulphorhodamine-B (SRB), ethidium bromide, phospate buffered saline (PBS), triton X-100, acridine orange, olive oil, haematoxylin and eosin were procured from Sigma-Aldrich Co. LLC (St. Louis, MO, USA). Chang cell lines were procured from the National Centre for Cell Sciences (Pune, MH, India) and cultured in DMEM medium supplemented with 5% (v/v) fetal bovine serum in a humidified incubator containing 5% CO_2_ and 95% air at 37°C. Only cells in the exponential growth phase were used for experiments.

### Preparation and standardization of the aqueous extract

The bark of the plant, *Terminalia paniculata* was collected from Manipal, Karnataka, India. It was authenticated (Voucher specimen: MCOPS/PHCOL/2009/2 deposited at Herbarium, Manipal College of Pharmaceutical Sciences, Manipal University, Manipal, Karnataka, India) and water extract (TPW) was prepared according to previously established methods [4]. Then, using an established HPLC protocol, the extract was standardized by comparing the retention time and UV spectra of the chromatographic peaks with those of the reference standards as previously reported [4].

### *In vitro* hepatoprotective activity using Chang liver cells

#### Cytotoxicity based assays

The cytotoxic effect of TPW was measured using MTT (3-(4,5-dimethylthiazol-2-yl)-2,5-diphenyltetrazolium bromide) and sulphorhodamine-B (SRB) assays [9,10].

In MTT assay, exponentially growing cells were seeded in 96-well plates (10^4^ cells/well in 100 μl of medium) and keep overnight for 24 h at 37°C in CO_2_ incubator. Test solutions were prepared prior to the experiment by dissolving in 0.2% DMSO and diluted with the media. The cells were then exposed to different concentrations of extract (50–500 μg/ml; 100 μl/well). Cells in the control wells received the amount of medium containing 0.2% DMSO. After 48 h, media was removed and 100 μl of MTT stock solution (1 mg/ml in sterile PBS, pH 7.4) was added and incubated for another 4 h at 37°C. The assay is based on the reduction of a tetrazolium salt to coloured formazan product by mitochondrial dehydrogenase in viable cells. The formazan crystals in each well were dissolved in 100 ml of DMSO, the absorbance read at 540 nm on a scanning multi-well plate reader (ELx800, BioTek Instruments Inc., Winooski, VT, USA).

In SRB assay, cells were seeded and treated with different concentrations of extract as in MTT assay. After 48 h, 50 μl of ice cold 30% TCA was added to each well of the plate (for fixing adherence cells) and incubated at 4°C for 1 h. Later, the wells were washed with distilled water (minimum 4 times). Next, 50 μl of 0.05% w/v (in 1% acetic acid) sulphorhodamine-B (SRB) solution was added to each well and the plate incubated for 30 min in dark conditions. The plate was then rinsed with 1% acetic acid (minimum 4 times) to removed unbound dye and dried at room temperature. Finally, 10 mM Tris base was added to each well to solubilize the protein bound dye. Absorbance was read at 540 nm on a scanning multi-well plate reader (ELx800, BioTek Instruments Inc., Winooski, VT, USA).

The percentage of growth inhibition for both MTT and SRB assays was calculated using the following formula:

$$\begin{array}{l} \%\;\mathrm{cytotoxicity}=\left[\left(A_{C}-A_{B}\right)-\left(A_{T}-A_{B}\right)\right]/\left(A_{C}-A_{B}\right) \end{array}$$

where A_C_, A_T_ and A_B_ is absorbance of control, test and blank, respectively.

### *In vivo* toxicological evaluation

Acute and sub-chronic toxicity was assessed according to the guidelines of Organisation for Economic Cooperation and Development (OECD) and principles of Good Laboratory Practice (GLP). Male Swiss albino mice (20–30 g) were used in experimental models as described below with the approval of the Institutional Animal Ethics Committee, Manipal University, Manipal, Karnataka, India (IAEC No. IAEC/KMC/51/2009-2010). They were housed in standard polypropylene cages, kept under ambient temperature (25–30°C) and relative humidity of 60–70% in a 12 h light–dark cycle. The animals were provided with a normal pellet diet (Amrit Feeds Ltd., Pune, MH, India) and water *ad libitum*.

#### Acute oral toxicity assay in mice

The acute oral toxicity study was conducted as per the OECD test guideline 420 (acute oral toxicity – fixed dose procedure). Twenty four Swiss albino mice of either sex were divided into four groups (n = 6) and were orally administered with a single dose of 300 mg, 1000 mg, 2000 mg, or 5000 mg/kg body weight (b.w.) of TPW extract. Animals were observed for possible behavioural changes such as tremors, convulsions, sleep, altered feeding, salivation, altered somato-motor activities and diarrhoea. These observations were continued for a period of 14 days, following which animals were sacrificed to examine gross changes to the vital organs.

#### Sub-chronic toxicity assay in mice

The sub-chronic oral toxicity study was conducted according to OECD guideline 407 (repeated dose 28-day oral toxicity study in rodents). Forty eight Swiss albino mice of either sex were divided into four groups (n = 12). Group I was orally fed with carboxymethyl cellulose (CMC; 0.5%) that served as control, whereas groups 2, 3 and 4 were orally administered with 750, 1500 and 3000 mg/kg of TPW extract, respectively. Previous reports from our laboratory showed that TPW extract at 200, 400 and 800 mg/kg did not cause adverse effects in rats [4].

Food and water intake of all the experimental groups were monitored daily at 09:00 hours. After 28 days of treatment, blood was collected from anaesthetized mice (ketamine-xylazine combination) by retro-orbital sinus puncture in EDTA-coated vials and plasma obtained by cold centrifugation (4°C) at 6000 rpm for 10 min. Thereafter, the animals were sacrificed by cervical dislocation and various vital organs were excised and weighed. Plasma sodium, potassium, calcium, aspartate aminotransferase (AST), alanine aminotransferase (ALT), acid phosphatase (ACP), alkaline phosphatase (ALP), urea, creatinine and total protein were assayed. All assay kits except total protein kit (Thermo Fisher Scientific Inc., Rockford, IL, USA) were obtained from the Roche Diagnostics India Pvt. Ltd., Mumbai, MH, India. Blood glucose was measured using glucometer (Accu-Chek Sensor, Roche Diagnostics India Pvt. Ltd., Mumbai, MH, India) in whole blood samples obtained from the tail vein.

### Evaluation of *in vivo* activity

#### Animals

Twenty four male Wistar albino rats (150–200 g) were used in experimental models as described below with the approval of the Institutional Animal Ethics Committee, Manipal Universty, Manipal, Karnataka, India (IAEC No. IAEC/KMC/51/2009-2010). They were housed in standard polypropylene cages, kept under ambient temperature (25–30°C) and relative humidity of 60–70% in a 12 h light–dark cycle. The animals were provided with a normal pellet diet (Amrit Feeds Ltd., Pune, MH, India) and water *ad libitum*.

#### Carbon tetrachloride (CCl_4_)-induced oxidative toxicity

This experiment was carried out according to previously described methods with slight modifications [8]. Rats were divided into four groups consisting of six animals in each group. Rats in group I (normal control) received distilled water containing 0.3% sodium carboxymethylcellulose (CMC-Na) (1 ml/kg body weight, p.o.) for 5 days. Group II (CCl_4_ control) received (CCl_4;_ S.D. Fine Chemicals, Mumbai, MH, India) 1:1 mixture of CCl_4_ and olive oil (1 ml/kg body weight, i.p.) on day 2 and day 3 along with 0.3% CMC-Na in distilled water. Group III was treated with the standard drug silymarin (100 mg/kg body weight, p.o.) daily for 5 days, and also received the CCl_4_–olive oil mixture (1:1, 1 ml/kg body weight, i.p.) on day 2 and 3, 30 min after administration of silymarin. Groups IV (test group animals) was administered a dose of 400 mg/kg (as this dose had earlier shown anti-inflammatory effect) body weight of TPW (p.o.) for 5 days. Additionally, 30 min after administration of TPW, they received a dose of the CCl_4_–olive oil mixture (1:1, 1 ml/kg, i.p.) day 2 and day 3. On day 7 (48 h after the last dose of TPW), animals were anaesthetized (ketamine-xylazine combination), blood was collected by retro-orbital sinus puncture, allowed to clot, and serum was separated for assessment of enzyme activity. The rats were then sacrificed by cervical dislocation; the livers were carefully dissected and cleaned of excess tissue. Part of the liver tissue was immediately transferred into 10% formalin for histopathological investigation.

#### Histopathological studies

Liver tissues were fixed in 10% formalin for at least 24 h, embedded in paraffin, and cut into 5 μm thick sections using a rotary microtome (RM2125, Leica Microsystems Inc., Bannockburn, IL, USA). The sections were stained with haematoxylin–eosin dye. A pathologist blind to the treatments performed the histological evaluation [11]. The photomicrographs of each tissue section were observed using CellˆA imaging software for laboratory microscopy (BX41TF, Olympus, Tokyo, Japan).

#### Biochemical determinations

Biochemical parameters were assayed according to standard methods. Activity of the following serum enzymes was measured: Alanine aminotransferase (ALT), aspartate aminotransferase (AST), and alkaline phosphatase (ALP) using automatic analyzer (Cobas C111, Fritz Hoffmann-La Roche Ltd., Basel, Switzerland). Total bilirubin was measured by the standard method [12]. Assay kits were obtained from Roche Diagnostics India Pvt. Ltd., Mumbai, MH, India.

Liver samples were dissected out, immersed in buffer, stored at −70°C. After freezing, homogenates (5% w/v) were prepared and centrifuged at 1000 rpm for 10 min using a refrigerated centrifuge (MIKRO 22R, Andreas Hettich GmbH & Co. KG, Tuttlingen, Germany). The supernatant was used for the estimation of glutathione (GSH), malondialdehyde (MDA) hydroperoxides, superoxide dismutase (SOD) and catalase levels [13].

##### Mitochondrial isolation

Mitochondria were isolated from rat liver as previously described [13]. In brief, the tissue was manually homogenized by four strokes with a Teflon pestle in solution I [230 mM mannitol, 70 mM sucrose, 1 mM EGTA and 5 mM HEPES (pH 7.4)] on ice. After centrifugation (1000 g for 80 s at 4°C), the supernatant was layered in solution II [460 mM mannitol, 14 mM sucrose, 1 mM EGTA and 10 mM HEPES (pH 7.4)] and centrifuged at 20000 g for 5 min at 4°C. The mitochondrial pellet was resuspended in 215 mM mannitol, 71 mM sucrose, 10 mM succinate and 10 mM HEPES (pH 7.4), and kept on ice until the mitochondrial staining procedure was performed.

##### Isolated mitochondrial staining

Isolated mitochondrial preparation was stained with help of JC-1 (5,5’,6,6’-tetrachloro-1,1’,3,3’-tetraethylbenzimidazol-carbocyanine iodide) dye (Sigma-Aldrich Co. LLC, St. Louis, MO, USA). The concentration of mitochondrial preparation was diluted to 40 μg/ml and used for staining. Final concentration of JC-1 staining solution was 0.2 μg/ml. 90 μl of JC-1 staining solution was added to 10 μl of isolated mitochondrial sample and an excitation wavelength of 490 nm and an emission wavelength of 590 nm were used to visualize the samples with help of inverted microscope with fluorescence attachment (Eclipse TS100, Nikon Instruments Inc., Melville, NY, USA).

### Cell culture studies

#### Apoptosis assay

The following experiment, modified from a previously described protocol [14], was employed to elucidate the mechanism of protection offered by TPW against CCl_4_-induced toxicity. Chang liver cells were cultured in DMEM supplemented with 10% FBS, in a humidified atmosphere containing 5% CO_2_ at 37°C. A monolayer of exponentially growing cells was harvested using trypsin-EDTA solution and cell suspensions were prepared for experiments. The following groups were employed.

Group 1 – Normal control: Cells not treated either with TPW or CCl_4_.

Group 2 – Drug control (TPW alone): Cells treated with TPW (100 μg/ml) for 30 min.

Group 3 – CCl_4_ control (CCl_4_ alone): Cells exposed to CCl_4_ [0.4% (v/v) CCl_4_ in 0.25% DMSO prepared in serum free culture medium].

Group 4 – TPW + CCl_4_: Cells treated with TPW (25, 50 and 100 μg/ml) for 30 min before treatment with CCl_4_.

Chang liver cells (2 × 10^6^) were grown in sterile 10 cm diameter tissue culture plates, treated according to experimental design and harvested to prepare the lysate.

•After CCl_4_ exposure, cells were harvested under non-denaturing conditions, medium was removed and cells were rinsed once with ice-cold PBS.

•PBS was removed and 0.5 ml ice-cold 1× cell lysis buffer plus 1 mM phenylmethylsulfonyl fluoride (PMSF) was added to each plate and incubated on ice for 5 min.

•Cells were scraped off the plate and transferred to appropriate tubes.

•Lysates were sonicated on ice, microcentrifuged for 10 min at 4°C and the supernatant transferred to a new tube.

•The supernatant was collected as the cell lysate. Single-use aliquots were stored at −80°C.

The markers were assessed using PathScan® Apoptosis Multi-Target Sandwich ELISA kit (#7105, Cell Signaling Technology Inc., Danvers, MA, USA). The protocol was followed as per the standard procedure supplied by the manufacturer of the kit. This method was employed to analyze the expression of apoptosis associated proteins such as phospho-p53, p53, cleaved caspase-3, phospho-Bad, Bad and cleaved PARP and evaluate the protection offered by TPW in Chang liver cells.

### Statistical analysis

All results were reported as mean ± S.E.M. (n = 6). The data were analysed using Prism 5.03 Demo Version (GraphPad Software Inc., La Jolla, CA, USA) by one-way analysis of variance (ANOVA), and statistically significant effects were further analysed by comparing means using Dunnett’s post hoc test. Statistical significance was considered at *p* < 0.05.

## Results

### Standardization of TPW with respect to its constituent polyphenols

**Figure 1:**
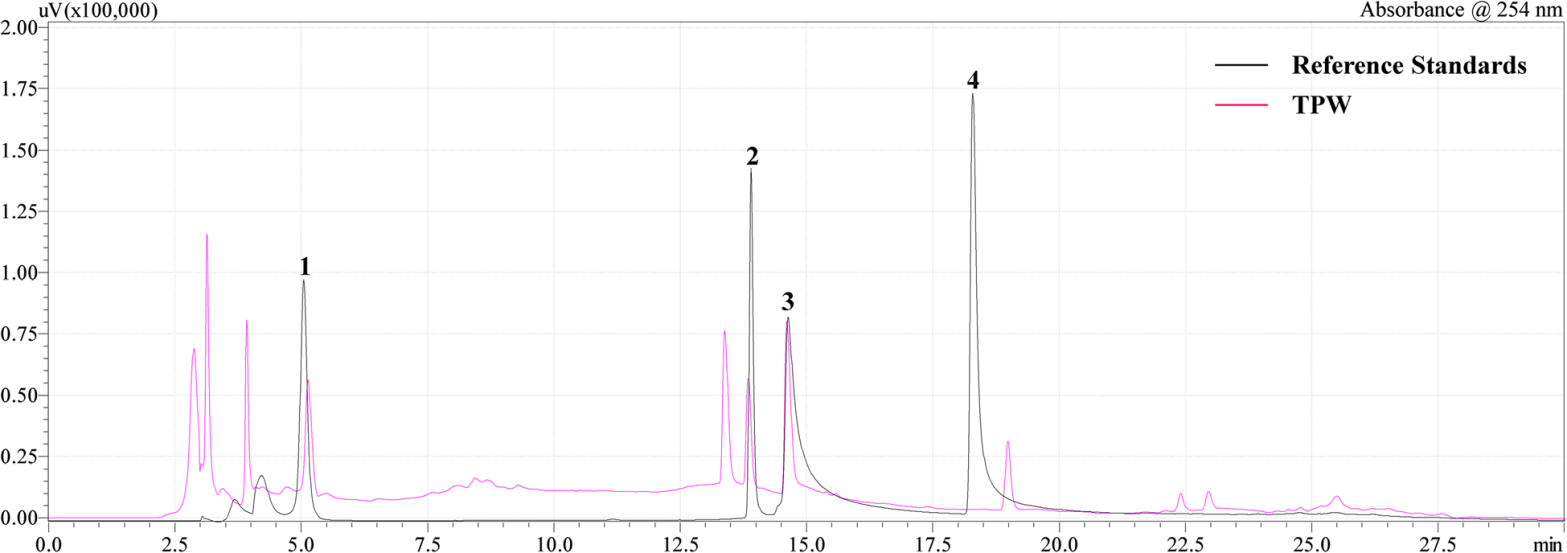
**Analytical HPLC-chromatogram of an aqueous bark extract of *Terminalia paniculata *at 254 nm.** (1) Gallic acid; (2) Rutin; (3) Ellagic acid; (4) Quercetin. The chromatographic peaks of the analytes were confirmed by comparing their retention time (Rt) and UV spectra with those of the reference standards (≥ 97% purity).

Standardization of TPW, used in the current study, confirmed the presence of polyphenols as shown in Figure 1. viz., gallic acid (0.68 ± 0.07 μg/mg), rutin (0.49 ± 0.05 μg/mg), ellagic acid (0.61 ± 0.06 μg/mg) and quercetin (0.19 ± 0.03 μg/mg) which were in accordance with results reported earlier [4].

### *In vitro* cytotoxicity assays

The IC_50_ of TPW in the MTT and SRB cytotoxicity assays performed on Chang liver cell lines was found to be 535.75 ± 15.59 μg/ml and 541.26 ± 22.44 μg/ml, respectively. As the values are high (>500 μg/ml), it indicates TPW does induce toxicity in the normal liver cells.

### Toxicological assessment

#### Acute toxicity studies in mice

No mortality was observed in animals that were orally administered up to 5000 mg/kg of extract. There were also no adverse behavioural changes, diarrhoea, salivation or food aversion. There was no major change in the gross weight of vital organs (data not shown).

#### Sub-chronic toxicity studies in mice

There were no significant alterations in weight gain, food and water intake and mean organ weight to body weight ratio of control and TPW treated groups (Tables 1 and 2). Sub-chronic doses of TPW extract showed no significant effect on plasma contents of electrolytes, glucose, urea, creatinine, total protein and activity levels of various hepatic enzymes, viz. ACP, ALP, AST and ALT up to a dose of 3000 mg/kg body weight (Tables 3 and 4).

**Table 1:** **Effect of sub-chronic (28 days) administration of aqueous extract of *Terminalia paniculata *on body weight, food intake and fluid intake in Swiss albino mice**

**Groups**	**Body weight change (g)**	**Food utilization (weight gain/food intake)**	**Water intake (ml/day)**
**NC**	4.63 ± 0.17	1.697 ± 0.11	7.95 ± 0.78
**TPW 750**	4.91 ± 0.12^ns^	1.795 ± 0.13^ns^	8.01 ± 0.85^ns^
**TPW 1500**	4.84 ± 0.16^ns^	1.772 ± 0.10^ns^	7.91 ± 0.69^ns^
**TPW 3000**	5.01 ± 0.08^ns^	1.729 ± 0.11^ns^	8.14 ± 1.01^ns^

*TPW* aqueous extract of *Terminalia paniculata*, *NC* normal control.

*ns* no significance between TPW versus NC.

**Table 2:** **Effect of sub-chronic (28 days) administration of aqueous extract of *Terminalia paniculata *on organ to body weight ratio of Swiss albino mice**

**Groups**	**Organ to body weight ratio**						
	**Brain**	**Heart**	**Lungs**	**Liver**	**Spleen**	**Kidney**	**Adrenal**
**NC**	1.96 ± 0.14	0.53 ± 0.05	0.81 ± 0.08	5.44 ± 0.48	0.47 ± 0.06	1.68 ± 0.10	0.06 ± 0.005
**TPW 750**	1.88 ± 0.17^ns^	0.51 ± 0.07^ns^	0.76 ± 0.05^ns^	5.69 ± 0.51^ns^	0.49 ± 0.05^ns^	1.60 ± 0.13^ns^	0.08 ± 0.004^ns^
**TPW 1500**	2.05 ± 0.20^ns^	0.50 ± 0.06^ns^	0.73 ± 0.09^ns^	6.02 ± 0.43^ns^	0.50 ± 0.04^ns^	1.57 ± 0.11^ns^	0.09 ± 0.004^ns^
**TPW 3000**	1.91 ± 0.16^ns^	0.49 ± 0.04^ns^	0.79 ± 0.06^ns^	5.87 ± 0.47^ns^	0.52 ± 0.05^ns^	1.59 ± 0.15^ns^	0.07 ± 0.003^ns^

*TPW* aqueous extract of *Terminalia paniculata*, *NC* normal control.

*ns* no significance between TPW versus NC.

**Table 3:** **Effect of sub-chronic (28 days) administration of aqueous extract of *Terminalia paniculata *on plasma electrolyte levels, total protein and blood glucose in Swiss albino mice**

**Groups**	**Glucose (mg/dl)**	**Total protein (g/dl)**	**Sodium (mmol/L)**	**Potassium (mmol/L)**	**Calcium (mmol/L)**
**NC**	98.45 ± 5.48	5.42 ± 0.65	84.15 ± 3.09	45.05 ± 2.54	13.06 ± 1.31
**TPW 750**	101.28 ± 6.05^ns^	5.69 ± 0.59^ns^	82.74 ± 3.67^ns^	42.13 ± 2.37^ns^	12.88 ± 1.04^ns^
**TPW 1500**	105.77 ± 6.09^ns^	5.81 ± 0.64^ns^	80.49 ± 3.91^ns^	41.91 ± 2.07^ns^	12.43 ± 0.96^ns^
**TPW 3000**	99.29 ± 5.86^ns^	5.90 ± 0.60^ns^	83.06 ± 3.35^ns^	44.40 ± 2.41^ns^	12.79 ± 1.25^ns^

*TPW* aqueous extract of *Terminalia paniculata*, *NC* normal control.

*ns* no significance between TPW versus NC.

**Table 4:** **Effect of sub-chronic (28 days) administration of aqueous extract of *Terminalia paniculata *on plasma urea, creatinine and hepatic enzyme levels in Swiss albino mice**

**Groups**	**Urea (mg/dl)**	**Creatinine (mg/dl)**	**ACP (U/L)**	**ALP (U/L)**	**AST (U/L)**	**ALT (U/L)**
**NC**	9.23 ± 0.85	0.97 ± 0.05	23.48 ± 1.57	12.75 ± 1.04	45.21 ± 2.10	27.66 ± 2.54
**TPW 750**	9.47 ± 0.93^ns^	0.89 ± 0.03^ns^	22.95 ± 1.21^ns^	12.40 ± 1.10^ns^	43.89 ± 1.86^ns^	26.90 ± 2.92^ns^
**TPW 1500**	9.70 ± 0.96^ns^	0.94 ± 0.04^ns^	23.28 ± 1.50^ns^	12.89 ± 0.86^ns^	42.09 ± 2.46^ns^	28.48 ± 2.73^ns^
**TPW 3000**	9.89 ± 1.01^ns^	1.02 ± 0.06^ns^	23.84 ± 1.33^ns^	13.02 ± 0.90^ns^	44.27 ± 1.78^ns^	27.36 ± 2.66^ns^

*TPW* aqueous extract of *Terminalia paniculata*, *NC* normal control, *ACP* acid phosphatase, *ALP* alkaline phosphatase, *AST* aspartate aminotransferase, *ALT* alanine aminotransferase.

*ns* no significance between TPW versus NC.

#### Histopathological observations

**Figure 2:**
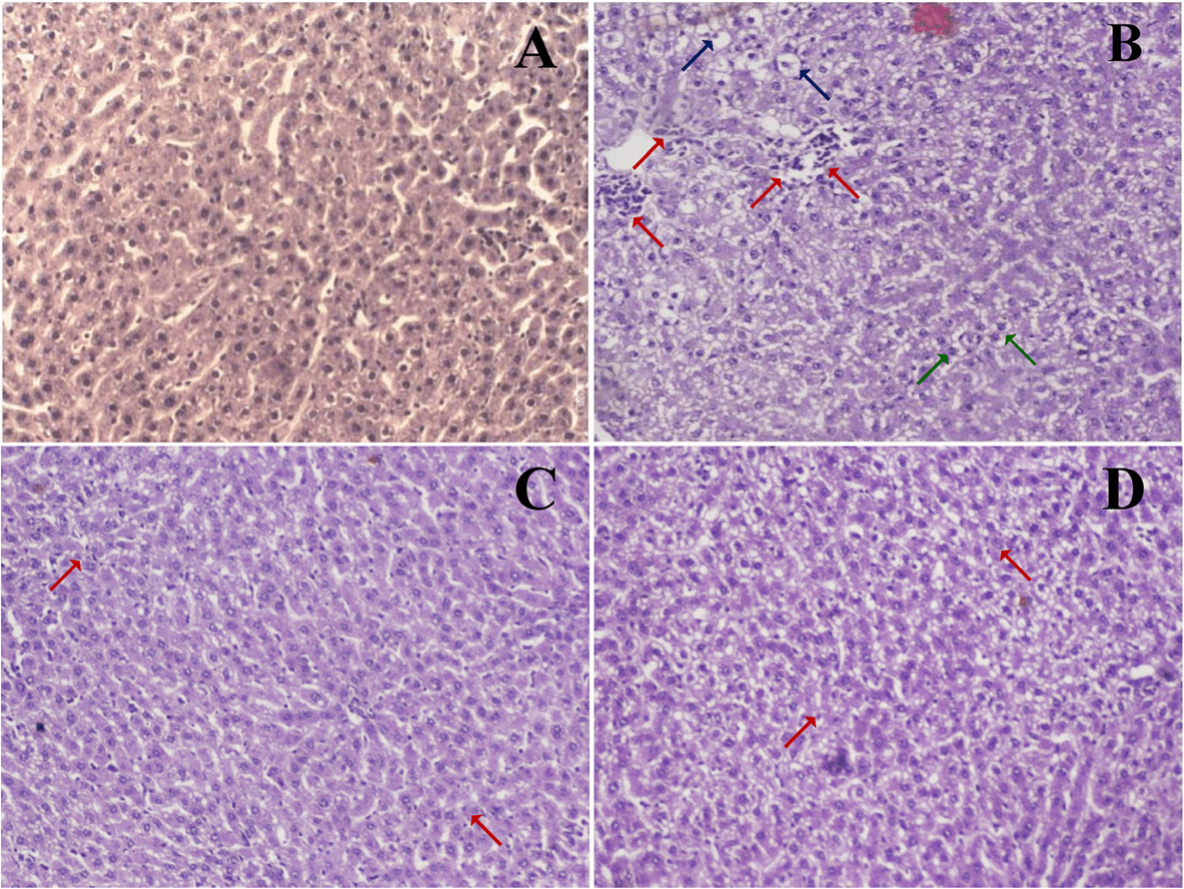
**Photomicrographs of histological changes of rat liver at magnification of 10×.** (**A**) normal control showed well maintained histology of rat liver (−); (**B**) CCl_4_ treated control rats showed lymphocyte infiltration (red arrow), fatty changes (blue arrow) and liver cell hypertrophy with congestion of hepatic sinusoids (green arrow) (+++); (**C**) Silymarin (100 mg/kg, p.o.) + CCl_4_ (1.0 ml/kg in olive oil 1:1, i.p.) treated group showed scarce lymphocyte infiltration and preserved cell architecture (+); (**D**) TPW (400 mg/kg, p.o.) + CCl_4_ (1.0 ml/kg in olive oil 1:1, i.p.) treated rat liver showed improved structure with mild lymphocyte infiltration (+). Scoring was performed as follows: –, no injury; +++, severe injury; ++, moderate injury; +, mild injury.

Histopathological observations of liver sections from the control group showed normal cellular architecture with distinct hepatic cells, sinusoidal spaces (Figure 2A). In contrast, the CCl_4_ group exhibited the most severe damage of any of the groups. The liver sections in this group showed fatty changes, necrosis, ballooning degeneration, broad infiltration of lymphocytes, and the loss of cellular boundaries (Figure 2B). The liver sections of the rats treated with silymarin and TPW (Figure 2C & D) showed a relatively normal lobular pattern with a mild degree of fatty change, necrosis, and lymphocyte infiltration that was more similar to the control group (Figure 2).

### *In vivo* evaluation

#### Serum enzymes in CCl_4_ treated rats

Since oxidative stress is one of the major contributors to hepatic dysfunction, preliminary antioxidant screening of the plant extracts, which will be further examined in hepatoprotective experiments *in vivo*, is considered to be necessary. Because the aqueous extract exhibits pronounced antioxidant activity, it was selected for further testing in the hepatoprotective model using silymarin as the reference.

AST is usually found in a diversity of tissues, including mainly liver, heart, muscle, kidney and brain. It is released into the serum when tissue is damaged by some insult. ALT is, by contrast, generally found mainly in the liver. ALT values are usually compared to the levels of other liver enzymes, such as ALP and AST, to help determine which form of liver disease could be present. On the other hand, serum bilirubin, a key pigment found in the bile, is considered a true test of liver function, as it reflects the liver’s ability to take up, process, and secrete bilirubin into the bile. Laboratory tests can differentiate between the “indirect” (unconjugated) bilirubin and “direct” (conjugated) bilirubin as it goes through the conjugation process in the hepatocytes. Therefore, the hepatoprotective activity was interpreted according to the increase/decrease in AST (aspartate aminotransferase), ALT (alanine aminotransferase) and total bilirubin levels (Table 5). Our results indicated that TPW possesses hepatoprotective activity as it caused a marked decrease in the levels of AST and ALT enzymes. In addition, there was a marked decrease in total bilirubin and ALT levels in both standard and test treatment groups (Table 5).

**Table 5:** **Effect of the aqueous extract of *Terminalia paniculata *and silymarin on the hepatic enzyme activities in CCl**_**4**_**-treated rats**

**Groups**	**AST (U/L)**	**ALT (U/L)**	**ALP (U/L)**	**BilT (mg/dl)**
**Normal control**	107.55 ± 4.65	49.05 ± 1.30	25.87 ± 3.52	0.10 ± 0.03
**CCl**_**4**_**control (CCl**_**4**_**1 ml/kg, i.p.)**	589.04 ± 10.12^#^	298.21 ± 10.55^#^	175.51 ± 6.84^#^	1.05 ± 0.12^#^
**Silymarin (100 mg/kg, p.o.)**	243.02 ± 8.80*	107.72 ± 8.24*	48.63 ± 15.30*	0.23 ± 0.11*
**TPW** **(400 mg/kg, p.o.)**	272.33 ± 26.8*	131.27 ± 5.31*	65.66 ± 5.24*	0.30 ± 0.10*

*TPW* aqueous extract of *Terminalia paniculata*, *AST* asparate transferase; *ALT* alanine transferase, *ALP* alkaline phosphatase, *BilT* – total bilirubin.

Data represented as mean ± S.E.M (n = 6), analysed by one-way ANOVA followed by Dunnett’s *post hoc* test. ^#^*p* < 0.05 as compared to normal control, *p < 0.05 as compared to CCl_4_ group.

#### Liver antioxidant enzymes levels in CCl_4_ treated rats

As GSH (assayed in units/mg of protein) plays an important role in the detoxification process; the hepatic GSH content was examined. Group II (CCl_4_) group had significantly decreased GSH concentration up to 35% (*p* < 0.05) compared with that of control (Group I). However, pre-treatment with TPW significantly recovered the CCl_4_-induced GSH depletion to 19.79 ± 1.65 (*p* < 0.05) (Table 6).

**Table 6:** **Effect of the aqueous extract of *Terminalia paniculata *and silymarin on the hepatic antioxidant enzyme activities in CCl**_**4**_**-treated rats**

**Groups**	**GSH**	**CAT**	**TBARS (MDA)**	**Total thiol**	**GST**
**Normal control**	22.27 ± 1.15	12.98 ± 0.90	68.99 ± 2.51	14.11 ± 1.03	38.79 ± 2.34
**CCl**_**4**_**control (CCl**_**4**_**1 ml/kg, i.p.)**	14.45 ± 0.84^#^	2.91 ± 0.99^#^	159.99 ± 16.61^#^	7.63 ± 0.14^#^	14.04 ± 1.48^#^
**Silymarin (100 mg/kg, p.o.)**	21.09 ± 1.85*	10.32 ± 1.28*	76.97 ± 8.49*	12.26 ± 0.51*	23.47 ± 1.85*
**TPW (400 mg/kg, p.o.)**	19.79 ± 1.65*	8.77 ± 1.31*	79.00 ± 5.93*	11.15 ± 0.96*	20.07 ± 3.33*

TPW – aqueous extract of *Terminalia paniculata*; GSH– glutathione (Unit/mg of protein); CAT – catalase (Unit/mg of protein); TBARS/MDA – thiobarbituric acid reactive substances / malondialdehyde (nMoles/mg of protein); Total thiol (Unit/mg of protein); GST – glutathione-S-transferase (μMole GSH-CDNB formed per mg of protein).

Data represented as mean ± S.E.M (n = 6), analysed by one-way ANOVA followed by Dunnett’s *post hoc* test. ^#^*p* < 0.05 as compared to normal control, *p < 0.05 as compared to CCl_4_ group.

The concentrations of other hepatic antioxidant markers such as total thiols, catalase and GST were significantly decreased in the CCl_4_ group (*p* < 0.05), when compared with the control group. On the other hand, pre-treatment with TPW produced a significant increase (*p* < 0.05) in levels of total thiols (11.15 ± 0.96 Unit per mg of protein), catalase (8.77 ± 1.31 Unit per mg of protein) and GST (20.07 ± 3.33 μMole GSH-CDNB formed per mg of protein) (Table 6). However, MDA (nmoles/mg of protein) levels were significantly (*p* < 0.05) decreased after TPW pre-treatment as compared to the CCl_4_ group (Table 6).

#### Mitochondrial staining in isolated hepatic cells

**Figure 3:**
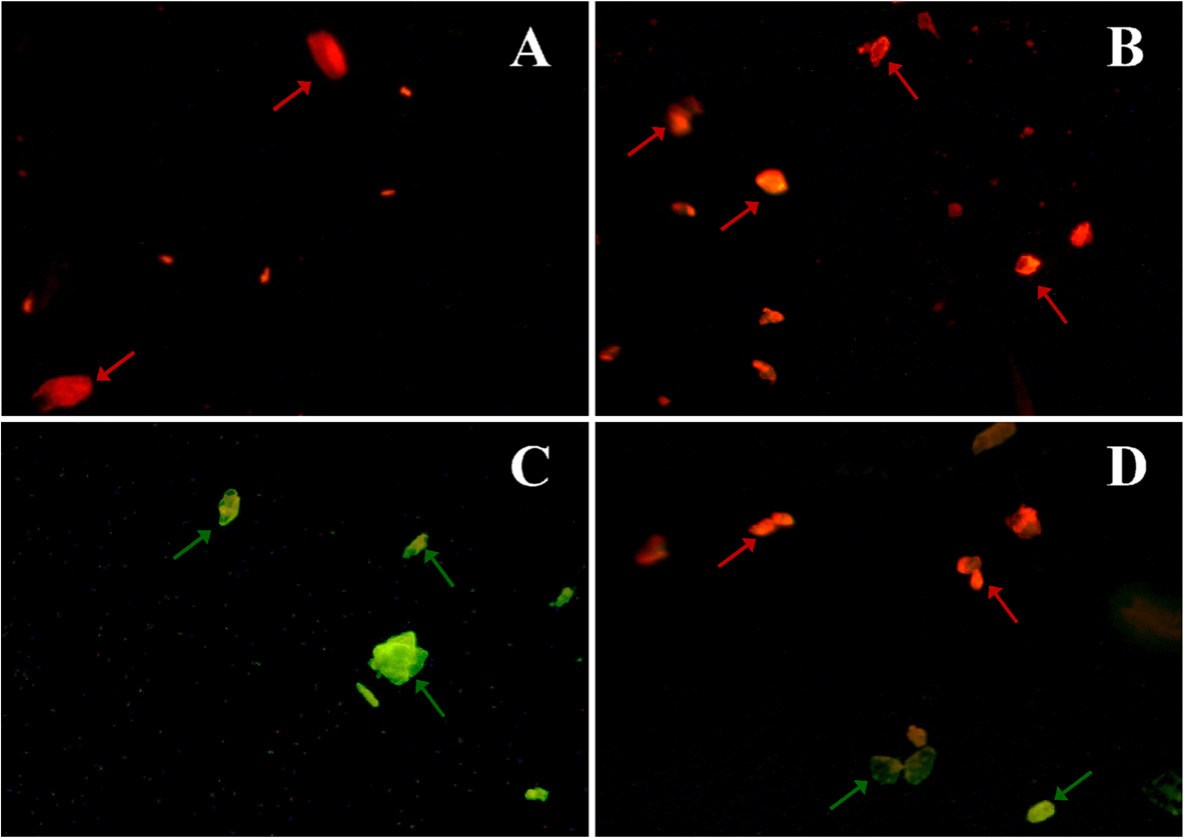
**Photomicrographs of isolated mitochondrial (stained with JC-1 dye) at magnification of 40×.** (**A**) Normal liver, the dye JC-1 concentrates in the matrix and bright red fluorescence was observed; (**B**) TPW control (400 mg/kg) showed similar features as compared to normal liver; (**C**) CCl_4_ treated control liver showed a shift from red to green fluorescence on incubation with JC-1 dye, which indicates damage to the inner mitochondrial membrane (green arrow); (**D**) TPW (400 mg/kg) + CCl_4_ treated liver showed red and mild green fluorescence up on incubation with JC-1 dye, which indicates mitochondrial inner membrane integrity was maintained.

Mitochondrial fractions were prepared from the liver of rats from the normal group, the CCl_4_ treated groups and the TPW treated group (Figure 3). The mitochondrial inner membrane potential was studied from the uptake of the cationic carbocyanine dye, JC-1 into the mitochondrial matrix. In the normal group, the dye concentrated in the matrix and bright red fluorescence was observed. In CCl_4_ treated group, a shift from red to green fluorescence was observed which indicates damage to the inner mitochondrial membrane. This prevents the accumulation of the JC-1 dye in the mitochondrial matrix. In the TPW (400 mg/kg) treated group, red and mild green fluorescence was observed which indicates that the mitochondrial inner membrane integrity was maintained.

### Cell culture studies

#### Effect of TPW on CCl_4_-induced apoptotic signaling proteins in Chang cells

The levels of apoptotic markers were significantly elevated in CCl_4_-treated Chang liver cells. TPW pre-treatment significantly attenuated the levels of phospho-p53, p53, cleaved caspase-3, phospho-Bad, Bad and cleaved PARP (Table 7). TPW, *per se,* did not produce any change in apoptotic markers compared to the normal control.

**Table 7:** **Effect of aqueous extract of *Terminalia paniculata *on apoptotic markers in CCl**_**4**_**-treated Chang liver cells**

**Groups**	**Phospho-p53**	**p53**	**Cleaved caspase-3**	**Phospho-Bad**	**Bad**	**Cleaved PARP**
**Normal control**	0.134 ± 0.037	0.129 ± 0.020	0.126 ± 0.023	0.141 ± 0.024	0.146 ± 0.040	0.135 ± 0.042
**TPW (50 μg/ml) control**	0.130 ± 0.025	0.131 ± 0.028	0.123 ± 0.020	0.136 ± 0.033	0.152 ± 0.059	0.138 ± 0.066
**CCl**_**4**_**control**	1.896 ± 0.081^*^	2.069 ± 0.054^*^	2.182 ± 0.091^*^	1.758 ± 0.037^*^	2.367 ± 0.072^*^	2.211 ± 0.101^*^
**TPW (25 μg/ml) + CCl**_**4**_	1.384 ± 0.066^*,#^	1.514 ± 0.033^*,#^	1.487 ± 0.040^*,#^	1.324 ± 0.051^*,#^	1.868 ± 0.064^*,#^	1.445 ± 0.047^*,#^
**TPW (50 μg/ml) + CCl**_**4**_	1.193 ± 0.050^*,#^	1.148 ± 0.057^*,#^	1.075 ± 0.072^*,#^	1.219 ± 0.035^*,#^	1.644 ± 0.049^*,#^	1.117 ± 0.053^*,#^

All values are expressed as mean ± SEM and experiments were carried out in triplicate. ^*^p < 0.05 vs. normal control; ^#^*p* < 0.05 vs. CCl_4_ control. The levels of the apoptotic markers were expressed in terms of absorbance as per kit’s protocol.

## Discussion

In developing countries, the indigenous population largely depend on traditional systems of medicine. Plants have long been used for therapeutic purposes, and many of the currently available drugs are directly or indirectly derived from plants [6].

Preparations obtained from natural sources are known to contain natural toxic compounds and hence the TPW extract was subjected to a detailed toxicological evaluation as per Interagency Research Animal Committee (IRAC) and Organization for Economic Co-operation and Development (OECD) guidelines. Results obtained from the acute oral toxicity test conducted as per IRAC and OECD guidelines clearly indicates that the median lethal dose (LD_50_) of TPW extract is >5000 mg/kg, b.w. suggesting that TPW extract is nontoxic. These results also validate the consumption of *Terminalia paniculata* among the local populace of India as therapeutic agent according to the traditional system of medicine [3].

Phenolic compounds from plants have been reported to be responsible for antioxidant activity [8,15]. Previous studies from our laboratory have demonstrated the presence of phenolic compounds such as gallic acid, ellagic acid, rutin and quercetin in TPW [4]. In this study, the hepatoprotective effect of TPW was evaluated in Chang liver cells. This human liver cell line is considered an appropriate model to study *in vitro* toxicity in the liver since it retains many of the specialized functions which are characteristics of normal human hepatocytes [16].

Most of the previous information related to the antioxidant properties of TPW was generated in isolated *in vitro* systems. In order to obtain this information from living animals, we chose the hepatotoxicity model of rat intoxication with carbon tetrachloride (CCl_4_). A primary indication of hepatic damage induced by CCl_4_ was obtained by the evaluation of hepatic enzymatic markers of injury such as AST and ALT. The levels of AST and ALT, 48 h after the administration of CCl_4_, were significantly elevated relative to the control group. These enzymes enter the circulatory system due to altered permeability of membranes and their increased levels reflected severe damage to the structural integrity of the liver [17,18]. Administration of TPW significantly attenuated CCl_4_-induced elevation of AST and ALT, indicating its hepatoprotective activity.

It has been reported that CAT, GSH and GST constitute the mutually supportive defense against reactive oxygen species [19,20]. In the present study, we demonstrated that CCl_4_ led to a significant drop in the levels of antioxidant enzymes, namely CAT, GSH and GST, probably due to oxidative stress-induced protein inactivation [21]. TPW and silymarin were able to prevent CCl_4_- induced decay by exerting free radical scavenging effects. This effect was also observed at the histological level.

It is now generally accepted that maintenance of mitochondrial membrane potential is necessary for mitochondria to carry out their oxidative functions [22]. In the present work, the effect of TPW on liver mitochondrial membrane potential in CCl_4_ intoxicated rat was assessed. Treatment of rats with CCl_4_, damaged the liver mitochondria as characterized by the dissipation of the mitochondrial membrane potential, which is in agreement with previously published reports [13]. TPW could maintain the integrity of the mitochondrial membrane, which confirmed its protective effect through an antioxidant mechanism.

Mitochondrial membrane is involved intimately in the p53-mediated apoptotic pathway. Following DNA damage, p53 is phosphorylated and phospho-p53 translocates to the nucleus triggering multiple mechanisms that include modulation of Bcl-2, Bax and other proteins, amplification of death signals and activation of caspases [23]. In our study, we observed elevation of phospho-p53, p53, phospho-Bad, Bad, cleaved caspase-3 and phospho-PARP in CCl_4_-treated Chang cells. The sensitivity of cells to apoptotic stimuli depends on the balance between pro- and anti-apoptotic Bcl-2 proteins. Disruption in the normal function of the anti-apoptotic Bcl-2 proteins leads to formation of pores in the mitochondria through which cytochrome-C and other pro-apoptotic molecules are released [24]. This in turn leads to apoptosome formation and activation of the caspase cascade. This cascade eventually activates effector caspases, such as caspase-3 and caspase-6, that are responsible for the cleavage of the key cellular proteins, such as cytoskeletal proteins. This in turn causes the typical morphological changes that are observed in cells undergoing apoptosis [25]. In the present study, the elevated levels of caspase-3 in Chang cells with CCl_4_ treatment correlated with the events of mitochondrial damage as observed by JC-1 staining in isolated mitochondria from rat liver. We also observed changes in PARP, a protein involved in DNA repair. Caspase-3 inactivates PARP impairing its ability to repair damaged DNA, which was in accordance with the increased levels of phospho-PARP (inactive form) [26]. TPW pre-treatment reversed the changes in the levels of proteins assessed indicating its anti-apoptotic mechanism of action.

To summarize, TPW confers overall protection to the liver against oxidative damage induced by CCl_4_ by mechanisms underlying its free radical scavenging potential integrated with preservation of the endogenous antioxidant enzymes like GST, GSH, MDA and CAT. The mechanism described here is consistent with the pharmacological properties traditionally attributed to this plant [4].

The phytochemical characterization of this plant had been performed earlier and the presence of constituents such as gallic acid and ellagic acid has been reported [4]. The findings of the present study are in accordance with the phytochemical components present in the plant, as gallic acid and ellagic acid (major constituents) are proven hepatoprotective agents which act by maintaining specific cellular homeostasis. This contributes to their preventive mode of action and beneficial effects against oxidative insults by acting through an antioxidant response-linked mechanism [27,28]. The presence of other constituents like quercetin and rutin [4] help mediate the free radical scavenging ability of TPW, owing to the strong antioxidant potential of these flavonoids [29]. It is due to this property of these constituents that the hepatoprotective activity of the extract is mediated by the normalization of impaired membrane function.

## Conclusion

Our study demonstrates the preventive potential of *Terminalia paniculata* as a hepatoprotective agent. This effect can be attributed to the presence of flavonoids. Further studies elucidating these findings are needed to explore the mechanisms by which hepatoprotection is conferred. The study serves as an initial step towards this end.

## Abbreviations

CCl4: Carbon tetrachloride; TPW: Aqueous bark extract of *Terminalia paniculata* Linn; OECD: Organisation for economic cooperation and development; ACP: Acid phosphatase; ALT: Alanine aminotransferase; AST: Aspartate transaminase; ALP: Alkaline phosphatase; CAT: Catalase; GSH: Glutathione; GSH: Glutathione-S-transferase; MDA: Malondialdehyde; SOD: Superoxide dismutase; PBS: Phospate buffer saline; MTT: 3-(4,5-Dimethylthiazol-2-yl)-2,5-diphenyltetrazolium bromide; SRB: Sulphorhodamine-B; DMEM: Dulbecco’s modified eagle medium; FBS: Fetal bovine serum; EGTA: Ethylene glycol tetraacetic acid; HEPES: 4-(2-hydroxyethyl)-1-piperazineethanesulfonic acid; JC-1: 5,5’,6,6’-tetrachloro-1,1’,3,3’-tetraethylbenzimidazol-carbocyanine iodide.

## Competing interests

The authors declare that they have no competing interests.

## Authors’ contributions

ST, PGN, RS and KNK designed the study and were involved in the manuscript preparation. PB performed the extraction of the extract and its HPLC analysis. AK and PGN performed *in vitro* toxicity study while PB, RS and NK performed acute and sub-chronic toxicity studies of the extract. AK, ST, HVJ and PGN evaluated the extract in CCl_4_-treated in rats. AK and ST performed biochemical estimations and histopathology. HVJ and PGN estimated the apoptotic markers by kit based assay in Chang cells and performed mitochondrial isolation from rat liver tissues for JC-1 staining. All authors read and approved the final manuscript.

## Pre-publication history

The pre-publication history for this paper can be accessed here:

http://www.biomedcentral.com/1472-6882/13/127/prepub
